# The impact of anti-Asian racism on routine activities and mental health among Korean American older adults and their caregivers

**DOI:** 10.3389/fpubh.2023.958657

**Published:** 2023-02-22

**Authors:** Hae-Ra Han, Deborah Min, Ji-Young Yun, Jin Hui Joo, Hochang Benjamin Lee, Simona Kwon

**Affiliations:** ^1^The Johns Hopkins School of Nursing, Baltimore, MD, United States; ^2^The Johns Hopkins Bloomberg School of Public Health, Baltimore, MD, United States; ^3^New York University School of Medicine, New York, NY, United States; ^4^Massachusetts General Hospital, Boston, MA, United States; ^5^University of Rochester School of Medicine, Rochester, NY, United States

**Keywords:** anti-Asian racism, mental health, Korean American, older adults, caregiver

## Abstract

**Introduction:**

Reported anti-Asian discrimination has been on the rise since the COVID-19 pandemic. Nevertheless, limited research addresses the health impact of perceived anti-Asian racism on Asian Americans, especially among older adults, during COVID-19. To address the gap, we examined how the novel coronavirus pandemic affected Korean American older adults, one of the largest Asian subgroups. Specifically, this study addressed the magnitude of racism or discrimination related to the pandemic and impact of anti-Asian racism on negative mental health symptoms among Korean American older adults and their caregivers.

**Methods:**

We used survey data collected from 175 Korean American older adults with probable dementia and their primary caregivers (female = 62%, mean age = 71 years) who went through eligibility screening for an ongoing randomized controlled trial involving dyads in the Baltimore-Washington and the New York Metropolitan areas (ClinicalTrials.gov Identifier: NCT03909347).

**Results:**

Nearly a quarter of the survey sample reported they were fearful for their safety due to anti-Asian racism related to the pandemic. Additionally, 47% of the respondents indicated changes to routine activities due to anti-Asian racism or discrimination related to COVID-19. The most common changes included avoiding walking alone or physical activities outside, followed by avoiding public transportation or leaving the house to go to any public places such as grocery stores, churches, or schools, not carrying out usual social activities, and avoiding going to health care appointments. Multinomial logistic regression revealed that people who reported changes to routine activities were at least five times more likely (adjusted odds ratio = 5.017, 95% confidence interval = 1.503, 16.748) to report negative mental health symptoms than those who did not. Being fearful for their own safety was not associated with experiencing negative mental health symptoms in the survey sample.

**Discussion:**

Study findings indicate that the increased reporting of anti-Asian racism during the COVID-19 pandemic has substantially affected Korean American older adults and their caregivers. The mechanism by which changes to routine activities is related to negative mental health symptoms is unclear, future research is needed to elucidate this pathway. Furthermore, our findings highlight the importance of identifying multi-level strategies to raise awareness of and to mitigate the reported surge of racism.

## Introduction

Anti-Asian discriminatory incidences encompassing verbal harassment, shunning (or deliberate avoidance), physical assault, and civil rights violations (e.g., refusal of service, workplace discrimination) have been on the rise since the Coronavirus disease 2019 (COVID-19) pandemic, with women reporting more hate incidents than men (1, 2). President Joe Biden signed the COVID-19 Hate Crimes Act in May 2021 (3). This was legislation to expedite the United States Justice Department reviews of anti-Asian hate crimes by making the reporting of hate crimes more accessible while ensuring reporting resources (3). Nevertheless, the aggregated reports by STOP AAPI Hate, the first ever attempt to systematically capture anti-Asian discrimination and xenophobia, by the Asian Pacific Policy and Planning Council showed more than 9,000 racially motivated attacks related to COVID-19 during March 2020-June 2021 (4).

Evidence supports the link between racism and negative health consequences, particularly among people of color (5). For example, the relative risk of COVID-19 death in the early pandemic was more than 10 times higher for Black and Hispanic/Latinx individuals younger than 50 years old compared to age-matched Whites in Illinois, attributable to limited access to healthcare and overall poorer population health resulting from structural racism (6). Despite the latest iteration of anti-Asian racism fueled by COVID-19, research addressing its impact on Asian Americans has been limited.

Available studies related to anti-Asian racism during COVID-19 examined adjustment and mental health problems among Chinese adolescents and their parents (7), affective reactions (e.g., fear, anxiety, depression, and avoidance) among Asian and non-Asian young adults (8), workplace experiences among Asian and non-Asian employees (9), race-based stress reported by news media coverage on COVID-19 related anti-Asian incidents or sentiments (10), racism-related social media use and depression among Asian Americans (11), or symptoms of depression and anxiety among young and middle-aged Asian Americans (12). None examined the impact of racism on older Asian Americans when, in fact, Asian Americans aged 45+ years had higher COVID-19 attributable mortality compared to non-Hispanic Whites (13, 14).

To address the gap in the literature, we conducted a survey and investigated how the COVID-19 pandemic was affecting Korean American lives. Korean Americans are the fifth largest subgroup among Asian Americans who are the fastest-growing racial group in the United States (15, 16). This study specifically examined the magnitude of racism or discrimination related to COVID-19 experienced by Korean Americans and how anti-Asian racism was associated with mental health among Korean American older adults and their caregivers.

## Materials and methods

### Design and sample

This was a secondary analysis of COVID-19 survey data collected from potential participants undergoing eligibility screening for an ongoing community-based randomized controlled trial—PLAN: Dementia Literacy Education and Navigation for Korean Elders with Probable Dementia and Their Caregivers (ClinicalTrials.gov Identifier: NCT03909347). Briefly, the primary goal of the PLAN trial is to test the effectiveness of the intervention, which consists of dementia literacy education and phone counseling with navigation assistance delivered by trained community health workers, on linkage to care for formal dementia evaluation among Korean American older adults with probable dementia.

The PLAN trial sample is dyad-based and consists of both Korean American older adults with probable dementia and their caregivers. Eligibility criteria for older adults include: (1) self-identified as first-generation Korean American; (2) ages 65 years or older; (3) Clinical Dementia Rating (CDR) 1.0+; (4) has a caregiver who lives in the same household or has at least weekly interactions; (5) resides in either the greater Baltimore-Washington metropolitan (i.e., Maryland, District of Columbia, and Northern Virginia) or the New York metropolitan areas (New York and New Jersey); and (6) able to consent or has a proxy available for consent. For caregivers, eligibility criteria include: (1) age 18 years or older; and (2) able to read and speak Korean.

The PLAN trial has two screening phases: (1) Mini-Mental Status Exam (MMSE) and (2) CDR. Once the first phase screening meets the pre-established criterion (MMSE < 24), the dyad is invited to a CDR interview. The COVID-19 survey was conducted for Korean American older adults who scored 24 or higher on the MMSE (i.e., normal cognitive function). For the older adults whose MMSE score was < 24, his/her caregiver was asked to participate in the COVID-19 survey. The study team approached 505 Korean American older adults and their caregivers. Among them, 220 (44%) agreed to participate and were scheduled for the COVID-19 survey; 45 were unable to participate. As a result, a total of 175 participants completed the study survey (85% older adults and 15% caregivers).

### Procedures

All study procedures were approved by the Johns Hopkins Medicine Institutional Review Board. COVID-19 survey data were collected between March and October 2021. Trained bilingual research staff collected survey responses mostly *via* phone which took on average about 20 min. Additional data collection methods involved sharing a link to an online survey through email or text (11 surveys or 6% of total surveys completed). These data collection methods coincided with COVID-related restrictions during the survey period. All data collection was done in Korean. No remuneration for participation was offered for this optional survey. Every participant provided verbal consent before completing the COVID-19 survey.

### Instrumentation

The study team developed the survey to better understand the impact of COVID-19 on Korean Americans' physical, emotional, and mental health. The 51-item survey also included questions about possible exposure to the virus, experiences with testing and treatment, and how one's life has changed as a result of COVID-19. In addition to sociodemographic questions such as age, sex, education, living arrangement, use of internet, use of social media, and study sites, key study variables included fear for safety (“*Are you fearful for your safety because of racism or discrimination related to COVID-19?”*), changes to routine activities (“*Have you changed any of the following activities because of potential racism or discrimination related to COVID-19?”)*, and Mental Health Impacts (MHI).

In particular, the MHI was adapted from the General Anxiety Disorder-7, Center for Epidemiological Studies Depression Scale, and the Impact to Event Scale-Revised and included items addressing nervousness, feeling depressed, feeling lonely, or having physical reactions when thinking about the experience with COVID-19 on a 4-point Likert scale (1 = Not at all or < 1 day a week to 4 = 5–7 days a week). An example question included, “Have you felt nervous, anxious, or on edge?” Higher scores indicated more frequent negative mental health symptoms, with an internal consistency reliability coefficient of 0.77 in the survey sample.

### Analysis

Data analysis was conducted in SPSS version 27. We used descriptive statistics such as means, standard deviations, frequencies, and percentages to summarize survey sample characteristics, while also addressing the prevalence of fear for safety and changes to routine activities related to potential racism or discrimination related to COVID-19. The Kolmogorov-Smirnov normality test indicated that the mental health data were not normally distributed (*p* < 0.001). We therefore created three groups addressing low, middle, and high negative mental health symptoms based on MHI scores across four items: 4 or lower (i.e., experiencing mental health symptoms none or < 1 day a week), 5–8 (experiencing mental health symptoms 1–2 days a week), and 9 or higher (experiencing mental health symptoms 3 or more days a week), respectively. We then performed a multinomial logistic regression to examine the association between fear for safety and changes to routine activities related to racism during COVID-19, and negative mental health symptoms. For the regression, we estimated adjusted odds ratio (aOR) and the respective 95% confidence intervals (CI). Statistical significance was determined at *p* ≤ 0.05 for all tests.

## Results

### Sample characteristics

Table 1 presents the descriptive characteristics of the survey sample. The sample were mostly in their 70s (mean = 71 years), female (62%), and highly educated (mean = 15 years of education). The majority of survey respondents resided in an individual home setting such as a single home, condo, or townhouse (70%). More than half (55%) of respondents were from the Baltimore-Washington metropolitan area. When asked about the use of internet and social network services (SNS), more than two thirds said they used internet fairly often or very often (71%), whereas only about one of five said they used SNS fairly often or very often (21%). As for racism or discrimination related to COVID-19, nearly a quarter of the respondents (24%) reported that they were fearful for their safety, and 47% indicated that they made changes to their routine activities. With respect to MHI, more than half of the survey sample reported that they experienced negative mental health symptoms 1–2 days/week (41%) or 3+ days/week (14%), whereas 45% of them had no or minimal (< 1 day/week) symptoms in the past week.

**Table 1:** Survey sample characteristics (*N* = 175).

**Variable**	**Mean (SD) or %**
Age (range = 40–90), years	71 (8)
Female	62
Education (range = 4–23), years	15 (3)
Live in single home/condo/townhouse	70
Reside in Baltimore-Washington^†^	55
Use internet fairly often/very often	71
Use social networking service (SNS) fairly often/very often	21
Fearful for safety due to racism	23
Changed routine activities due to racism	47
**Mental health impacts (range** = **4–15)**	
Low [4 or less (not at all or < 1 day/week)]	45
Middle [5–8 (1–2 days/week)]	41
High [9 or higher (3 or more days/week)]	14

^†^Study participants were recruited from either Baltimore-Washington or New York Metropolitan areas.

### Changes to routine activities due to racism or discrimination related to COVID-19

Table 2 presents the most common changes to routine activities due to racism or discrimination related to COVID-19. Of those who endorsed any changes to routine activities (*n* = 83), avoiding walking alone or physical activities outside was most frequently reported (73%), followed by avoiding public transportation (42%) or leaving the house to go to any public places such as grocery stores, churches, or schools (41%), not carrying out usual social activities (33%), and avoiding going to health care appointments (4%).

**Table 2:** Changes to routine activities due to racism related to COVID-19.

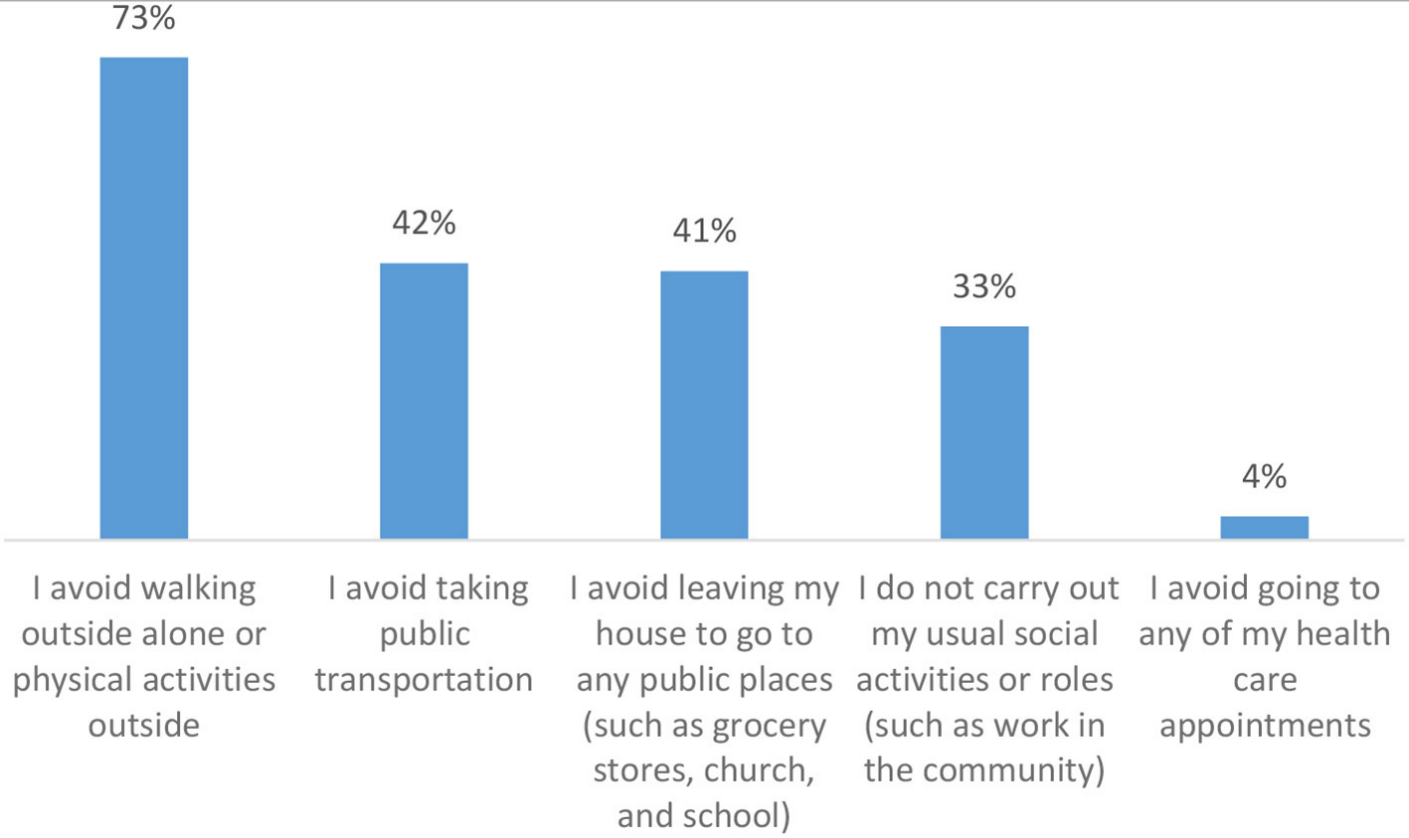

### Associations of racism related fear and changes to routine activities with mental health

Table 3 summarizes findings from the multinomial logistic regression models adjusted for age. Specifically, those who reported changes to routine activities due to COVID-19 related anti-Asian racism or discrimination had at least five times higher odds of reporting most frequent negative mental health symptoms than those who did not report any change (aOR = 5.017; 95% CI = 1.503, 16.748). Additionally, the odds of a person whose education level was high school or less being included in the high MHI scored group compared to the low MHI scored group was slightly more than four times higher than a person with some college or more education (aOR = 4.369; 95% CI = 1.303, 14.652). Having fear for safety related to anti-Asian racism was not significantly associated with mental health.

**Table 3:** Results from multinomial logistic regression models.

	**Middle MHI group**			**High MHI group**		
	**aOR**	**95% CI**		**aOR**	**95% CI**	
		**Lower**	**Upper**		**Lower**	**Upper**
Age	0.965	0.918	1.015	0.954	0.885	1.028
Male	0.801	0.395	1.626	0.429	0.120	1.530
Low education	1.259	0.530	2.990	4.369^*^	1.303	14.652
Residence in single home/condo/townhome	0.504	0.239	1.065	1.131	0.353	3.620
Greater Washington area	1.049	0.508	2.168	0.885	0.289	2.527
Little/no use of internet	1.050	0.468	2.356	1.364	0.423	4.405
Little/no use of SNS	1.514	0.624	3.674	1.848	0.444	7.693
Fearful for safety	0.596	0.242	1.467	1.591	0.517	4.898
Changed daily activities	1.265	0.615	2.604	5.017^**^	1.503	16.748

The reference categories for this analysis were: low MHI group, female, high education (some college+), residence in apartment, individuals in New York area, frequent use of internet, frequent use of SNS, no fear for safety, and no change in daily activities; models were adjusted for age. ^*^p < 0.05, ^**^p < 0.01.

## Discussion

Despite unequal burden of COVID-19 among Asian Americans coupled with ongoing anti-Asian racism related to COVID-19, research that investigates the impact of racism on older Asian Americans is scarce. This study investigated the impact of racism during COVID-19 and the relationship between anti-Asian racism and mental health among Korean Americans. The results revealed that racism against Asian Americans related to COVID-19 substantially affected Korean American older adults and their caregivers in the survey sample, with nearly half of them reporting changes in routine activities related to anti-Asian racism. Further, those who reported changes in routine activities had significantly increased odds of experiencing frequent negative mental health symptoms, suggesting the link between mental health and racism or discrimination related to COVID-19.

Our main finding of the significant association between changes in routine activities related to racism and negative mental health symptoms is consistent with the results reported in prior research involving people of color. Harmful effects of racism have been documented in a non-COVID-19 specific context with evidence supporting associated negative mental health outcomes. For example, a systematic review of studies involving Black American older adults (50+ years) revealed that racism was significantly associated with depressive symptoms, psychological distress, or anxiety in fourteen out of fifteen studies in which mental health measures were included (17). Another systematic review included longitudinal exposure of racial discrimination among children and found significant associations between accumulated racial discrimination and adverse health outcomes (e.g., substance use, risky sex behavior, disrupted cortisol slopes similar to traumatic stress) (18). Taken together, these findings indicate the significant negative impact of racism on health across the lifespan.

It is not completely clear why Korean Americans with changes in routine activities had more frequent negative mental health symptoms than those without. It might be that some of the changes caused by anti-Asian racism (e.g., avoiding usual social activities) aggravated the already substantially increased level of loneliness among older adults during the COVID-19 pandemic; loneliness has been associated with a range of physical and mental health problems including major depression (19). Another possibility is internalized racism. Internalized racism (i.e., a belief that one's racial group is inferior to other racial groups) has been suggested as a potential pathway through which racism may cause emotional and physical harm to minoritized people, though largely targeted Black Americans (20–22). According to a recent survey of Asian and Latinxs college students, Asian students endorsed higher levels of internalized racism and perceived a change in everyday discrimination compared to Latinxs during the COVID-19 pandemic (23). Similarly, an online sample of Asian Americans (mean age = 39 years) revealed that indirect racism experienced through family or friend was linked to compromised sleep quality and duration but the adverse effects of racism experience were lessened among those who reported high levels of ethnic/racial identity (24). The findings suggest the need for research addressing the mechanism through which racism influences diverse health outcomes among Asian Americans.

It is concerning to note that avoiding public transportation or going to public places such as churches or grocery stores was frequently reported among the study participants. Transportation is a key social determinant of health which disproportionately affects the most vulnerable groups in our society who often carry the highest health disparity burden such as non-English speaking immigrant older adults including Korean Americans (25). Additionally, given the central role of faith-based organizations in immigrant communities as epicenters for social, religious, and health promotion activities, the challenges and impact of these changes in routine activities such as avoiding public transportation resulting from fear of anti-Asian racism and xenophobia may have been insurmountable (26). Future research must thoroughly describe a wide range of health impacts caused by changes to these routine activities related to the increased reports of and incidences of anti-Asian racism during the COVID-19 pandemic, while identifying possible solutions to challenge ongoing xenophobia. Some of the suggested methods include social advocacy as well as education and training of students, public health professionals, and health agencies (27). Additionally, a national online survey of US adults (*N* = 1,141) conducted in March 2020 (28) indicated individuals who reported being more fearful of COVID-19 and who reported more misinformation about the virus and less trust in science had more negative attitudes toward Asians, suggesting the need for public awareness campaigns.

## Limitations

We recruited survey participants among those who were approached for an ongoing intervention trial designed to link Korean American older adults aged 65+ years to medical services for formal dementia evaluation. Due to the nature of this parent trial, it is possible that those who responded to this survey had poorer overall health status. In addition, this was a convenience sample hence the findings should be interpreted with caution. For example, the COVID-19 survey was taken in the early phase of the COVID-19 pandemic using virtual methods; that is, all who signed up for eligibility screening for the parent study were those who were able to follow our instructions to download and/or use a virtual videoconferencing tool, Zoom. These were often highly educated individuals (mean years of education = 15 years). Nationally, among Asian American older adults 65+, 43% had a bachelor's degree or higher (16+ years of education) in 2020 (29). Relatedly, more than half of the survey sample were recruited from the Baltimore-Washington metropolitan area. At the national level, anti-Asian hate crimes seem to occur more frequently in some of the major metropolitan areas such as New York. While such crimes increased by 189% across the country during the first quarter of 2021 compared to that of 2020, New York City saw a 262% increase during the same period (30). A recent analysis using geo-located tweet messages across the United States also revealed that New York was included in the top 10 geographical clusters with a higher proportion of hateful tweets against Asians related to COVID-19 (31). Finally, this study used cross-sectional data and we are unable to determine the temporality or causal direction between anti-Asian racism and adverse mental health symptoms. With the current study design, we are also unable to differentiate the influence of racism against Asians on mental health from the influence of the COVID pandemic itself. Future research using longitudinal designs may help unpack the long-term impact of anti-Asian racism related to the COVID-19 pandemic on mental health outcomes.

## Conclusion

This study showed that anti-Asian racism related to the COVID-19 pandemic was associated with Korean American older adults and their caregivers' change of routine activities. Such changes significantly affected Korean older adults, contributing to their experience of negative mental health symptoms. Though President Biden signed into law the COVID-19 Hate Crimes Act in May 2021, this is just a first step to recognizing anti-Asian racism as a national issue requiring expedited review, report, and response—however, there remains critical need for systems level changes (3). Asian Americans have experienced racism and xenophobia since the first wave of immigrants arrived to the United States in the mid-nineteenth century. Historically, anti-Asian hate crimes have been perpetuated by exclusionary and oppressive policies (32). Such hate crimes have surged during the COVID-19 pandemic and have revealed the absence of concrete resources, funds, and action needed to curb the problem including but not limited to better data reporting, disaggregating data to highlight the lack of research, resources for Asian Americans and Korean American groups, and policy changes to integrate Asian American history/studies in our education systems. Addressing historical and structural challenges that engender anti-Asian racism and xenophobia is essential to directing our society to set its course toward a more racially just and equitable one.

## Data availability statement

The raw data supporting the conclusions of this article will be made available by the authors, without undue reservation.

## Ethics statement

The studies involving human participants were reviewed and approved by Johns Hopkins Medicine IRB. Written informed consent for participation was not required for this study in accordance with the national legislation and the institutional requirements.

## Author contributions

H-RH originated the study, led the writing, and supervised the study. H-RH, DM, J-YY, JJ, HL, and SK contributed to the acquisition, analysis, or interpretation of data. H-RH, DM, and J-YY drafted the manuscript, and all authors contributed to the critical revision of the manuscript. All authors approved the final version of the manuscript.
